# Porcine Anti-viral Immunity: How Important Is It?

**DOI:** 10.3389/fimmu.2019.02258

**Published:** 2019-09-27

**Authors:** Kelly M. Lager, Alexandra C. Buckley

**Affiliations:** Virus and Prion Research Unit, United States Department of Agriculture, National Animal Disease Center, Agricultural Research Service, Ames, IA, United States

**Keywords:** swine, virus, disease, immunology, vaccine

## Abstract

Pork has become the number one meat consumed worldwide. Meeting the demand for pork has forced the revolution of swine production from traditional husbandry practices that involved a few pigs or small herds to intensive concentration of swine raised in multisite production systems. This dramatic change has made the production of pork very efficient, but it has also changed the ecology of many swine diseases, may encourage the emergence of new diseases, and amplifies the economic impact of swine diseases. Sustained treatment of diseases in livestock production is not feasible making prevention of disease a priority. Prevention of livestock diseases involves eliminating exposure to pathogens and anti-viral strategies to prevent or reduce clinical disease. For some swine diseases, efficacious vaccines can be made, however, for other diseases the host/pathogen relationship is more complex and efficacious vaccines are not available. Given the increasing demand for pork, the development of new approaches to improve swine anti-viral immunity is critical. Rate-limiting steps to improving vaccines are understanding how the pathogen interacts with the host's immune system, any immunopathology resulting from such interactions and how the host's immune system resolves the infection. Solving this puzzle will require sustained research and may require new technologies to battle contemporary diseases now wreaking havoc in swine production systems around the world. This Special Issue will focus on current swine viral diseases that are the most challenging to the global production of pork with contributions focusing on anti-viral immunity.

The quest for food is primal, and for most of mankind's existence, a daily struggle for all. About 15,000 years ago agriculture began to develop with the cultivation of crops and domestication of animals. This lessened the daily burden of searching for food and began providing time for creativity which has led to unimaginable technologic achievements, and to exponential growth in the human population. Despite all contemporary technology, the stability of our food supply is still vulnerable to many challenges that have existed since the beginning of agriculture, and to new ones that are associated with modern agriculture practices that produce massive amounts of food. Although, there are many challenges to meeting our absolute need for food, this review focuses on just one aspect of modern agriculture, the production of pork.

In the twenty first century, agriculture reflects a broad spectrum from subsistence farming to single-commodity production systems producing meat, milk, grains, fruits, and vegetables on an unprecedented scale. The increasingly efficient production of pork has made it more readily available and it has become the number one meat consumed worldwide (1). Meeting the demand for pork has forced the evolution of swine production from traditional inefficient husbandry practices involving a few pigs or small herds to an intensive concentration of swine that is divided into stages of production housed at different sites often far apart. This dramatic change has made the production of pork very efficient, but it has also changed the ecology of many swine diseases. Moreover, this style of production may encourage the emergence of new diseases, and amplifies their economic impact.

Control strategies for swine diseases are driven by the economic impact of the respective disease and the diseases can be divided into two broad categories: those that warrant interdiction and those that do not. There are many swine viruses that have been infrequently associated with disease; sometimes one or just a few animals are affected, other times the disease may spontaneously reach a high incidence in the herd, and then just as quickly, it dissipates. In general, these viruses can be economically insignificant to a regional or national swine herd and will not be further discussed. However, this group of viruses is still important since the reasons for when a sporadic viral disease might flare up in a herd, and then “burn out,” are poorly understood. It is still prudent to investigate the epidemiology of these “insignificant” viruses since they might 1 day become significant. Swine viruses causing, or that potentially could cause, significant economic disease in a herd are similar around the world and these viruses are the focus of this Special Topics review (Table 1). For a more comprehensive review of swine viral diseases, the reader is encouraged to read *Diseases of Swine*, 11th edition (2) and *Porcine Viruses: From Pathogenesis to Strategies for Control* (3). General information from these books about specific diseases is used extensively throughout this manuscript, and will not be cited repetitively.

**Table 1:** Swine viral pathogens of economic or zoonotic importance.

**Virus^**a**^**	**Economic^**b**^**	**Vaccine^**c**^**	**Zoonotic^**d**^**
ASFV	++++	No	–
FMDV	++++	Yes	–
CSFV	+++	Yes	–
ADV	++	Yes	–
PRRSV	++++	Yes	–
IVA-S	++	Yes	+++
PCV2	+	Yes	–
PEDV	+	Yes	–
SVA	+	No	–
JEV	+	Yes	++
HEV	+	No	++
Nipah virus	+	No	+++
EMCV	+	No	+
Menangle virus	+	No	+
VSV	+	No	+
VESV	+	No	+

a *ASFV, African swine fever virus; FMDV, foot and mouth disease virus; CSFV, classical swine fever virus; ADV, Aujeszky's disease virus; PRRSV, porcine reproductive and respiratory syndrome virus; IVA-S, influenza virus A- swine; PCV2, porcine circovirus type 2; PEDV, porcine epidemic diarrhea virus; SVA, Senecavirus A; JEV, Japanese encephalitis virus; HEV, Hepatitis E virus; EMCV, encephalomyocarditis virus; VSV, Vesicular stomatitis virus; VESV, Vesicular exanthema of swine virus*.

b *Economic impact ranging from + (infrequent/mild) to ++++ (frequent/severe)*.

c *Vaccine available to aid in control and prevention: yes or no*.

d *Zoonotic potential ranging from + (infrequent/mild) to ++++ (frequent/severe)*.

Successful pork production is dependent upon many variables of which animal health may be the most volatile. For example, just one biosecurity mistake in protocols designed to mitigate herd health risks can produce long-term health consequences for the herd and financial ruin for the producer. An economic loss of this nature for one producer can be exponentially amplified if such a mistake would cause a country to lose its export market. For example, about 25% of the US pork produced each year is exported making the US swine industry quite vulnerable since any pandemic of local or foreign origin could induce a ban on the importation of US pork for fear of transmitting the disease. Following the emergence of the 2009 influenza pandemic, such a ban or threat of a ban was enacted by more than 27 countries that produced an immediate loss of market. Although, it only took a few months to refute the ban, the industry still lost an estimated 1.5 billion dollars (4).

Vaccines can increase the resistance of an animal to infection making them an increasingly important tool in the health management of swine herds. Although, many vaccines are efficacious, continual pathogen mutation and the emergence of new virus threats drives a constant need for new and improved vaccines. Not surprisingly, many of the significant-economic-disease viruses are viruses for which there are no vaccine, or the current vaccine has limited efficacy. Any solution to the current swine vaccine problem will involve a better understanding of the pig's immune response to these pathogens with the hope of applying this knowledge toward the development and improvement of vaccines, and other more rapid responses to new diseases in the future.

Porcine reproductive and respiratory syndrome virus (PRRSV), a previously unknown swine virus, emerged in the late 1980s and spread around the world within a few years becoming the first of several swine pandemics that would occur over the next 30 years. PRRSV quickly became the number one health problem in major swine producing countries because it is able to affect all stages of production, is highly infectious, has a prolonged shedding duration, and perhaps most importantly, is able to dysregulate the pig's immune response. The economic impact of this virus is substantial, e.g., in 2013 PRRSV was estimated to cost just the US swine industry alone 660 M a year (5). A number of inactivated and attenuated vaccines are available in most countries with attenuated vaccines reported as superior to inactivated virus vaccines, suggesting the importance of the mucosal response for clinical protection. Despite being derived from various field viruses, attenuated virus vaccines are able to induce homologous protection, but only variable heterologous protection (6). The nature of this sometimes poor cross-protection is not understood and is a major obstacle to improving PRRSV vaccines.

Foot and mouth disease virus (FMDV) is a highly infectious virus that affects cloven-hooved animals causing vesicular lesions involving the feet and oral cavity resulting in a crippling disease and loss of production to the extent that this virus is the most important disease concern for livestock producers around the world. Once FMDV has been eradicated from a country, substantial resources are committed to keeping the country FMDV-free that include the regulated movement of livestock, substantial diagnostic testing, and the quest for safe and efficacious vaccines. Similar to PRRSV, FMDV vaccines are available, however, they are serotype specific and lack cross-protection among the 7 FMDV serotypes (7). Moreover, there can be variation within a subtype to a degree where the subtype-specific vaccine may not provide adequate protection. Although, a properly matched subtype vaccine can be used in control and eradication programs, it is prohibitively expensive to maintain a vaccine bank for all potential serotypes. There is a dramatic need for more cross-protective FMDV vaccines to help resolve FMDV epidemics when they occur, and maintain FMDV-free countries.

The control of influenza A viruses in swine (IAV-S) exemplifies the challenges of passively acquired maternal immunity in modern swine production. In the late 1990s, a novel IAV-S H3 subtype was first detected in the United States, and similar to the then endemic North American H1 subtype, the new virus caused respiratory disease in fattening hogs and sows (8). In response to this new H3 subtype lineage, specific inactivated vaccines were produced and combined with H1 vaccines to form new polyvalent vaccines that were efficacious in naïve older swine when administered prior to the onset of disease. However, as the intensification of swine production expanded, the ecology of IAV-S began to change with younger pigs becoming clinically affected which supported strategies to immunize the sows to provide passive immunity to their piglets. This was helpful, but the practice also jeopardized the ability to vaccinate young pigs to protect them against disease in later stages of production illustrating the passive immunity conundrum when using inactivated vaccines given intramuscularly to young pigs. Recently, an attenuated IAV-S vaccine has been released for sale in the US that is given intranasally to neonatal pigs to circumvent passively acquired immunity. It is reported to provide protection to young pigs and induce a broader protective immune response when compared to inactivated vaccines given intramuscularly (9).

IAV-S is a swine virus with zoonotic potential (Table 1). It is ubiquitous in swine producing regions around the world, and with variable frequency, swine-to-human transmission does occur. Fortunately, most transmission events are limited in scope of disease and subsequent human-to-human transmission. However, the 2009 influenza pandemic is a stark reminder of the potential for influenza viruses to jump species and become a pandemic. Reverse zoonosis, IAV transmission from people to swine, is being detected with similar frequency. This phenomenon is an important mechanism for transferring human viruses into swine where the virus can adapt to swine and be maintained as a potential reservoir of human-like IAV that could jump back to humans. Other zoonotic swine viruses are more regional in distribution and usually are not considered to cause significant economic loss. Crossover events with these viruses are much less frequent when compared to IAV-S; however, they can have a higher case fatality rate in people (Japanese encephalitis virus, Hepatitis E virus). Most zoonotic swine virus crossover events are very rare and typically cause minimal human disease, however, in 1998, Nipah virus, a previously unknown virus, emerged in swine in Malaysia. It caused a respiratory infection with high morbidity in all ages of swine on several farms. Sick pigs transmitted the virus to farm workers causing a severe illness with a Case Fatality Rate of about 40% in the initial outbreak (10). The disease was eradicated from the affected farms within months, and with the discovery that bats were the reservoir for this virus, precautions could be taken to prevent future infections which have been successful to date.

In 2013, porcine epidemic diarrhea virus (PEDV), one of the swine enteric coronaviruses that causes severe diarrhea in neonatal pigs, was discovered in the Western Hemisphere for the first time. Within 6 months of entry into the US, the virus had spread through all swine dense regions killing about 10% of the pig crop that year. Although, swine can mount a rapid protective immune response to PEDV, young pigs are unable to survive the disease long enough to develop a protective response which makes protecting young pigs the most important step to controlling this disease. The only option to protect the pigs is to provide passive maternal immunity to the suckling pig via colostrum/milk, a strategy that can work but is dependent on the sow having adequate exposure to PEDV to induce mucosal immunity. Although, wild-type PEDV might induce the best lactogenic and colostral immunity for the piglet, immunizing the sow herd with wild-type PEDV has serious safety concerns demonstrating the need for improved PEDV vaccines.

Beginning in late 2014 and into 2015, there were outbreaks of idiopathic vesicular disease in Brazil followed by similar outbreaks in the United States. Disease was most often recognized in market-weight swine and sows, and samples from these cases tested positive for Senecavirus A (SVA). Subsequently, the virus has also been identified in Canada, China, Colombia, and Thailand (11). Although, the clinical disease is mild in most animals, and the incidence is low when compared to most economically important swine diseases, vesicular lesions from SVA infection are indistinguishable from FMDV infection. Foot and mouth disease is the number one disease concern for livestock producers around the world which makes understanding the pathogenesis and immunology of any virus that may confound FMDV diagnostic investigations important. Recent SVA studies have demonstrated fulfillment of Koch's postulates, documented pathogenesis, and shown that contemporary isolates are closely related. Sterile protective immunity was demonstrated in piglets exposed to wild-type SVA and then challenged with the same isolate 7 weeks later (12). This preliminary research indicates pigs can develop a protective immune response and there is potential for the use of vaccines. There are still many questions about the ecology and epidemiology of this virus including (1) why these “mini-epidemics” occurred in different countries with similar viruses (over 94% nucleotide identity among contemporary isolates), (2) the apparent seasonality to the clinical expression of the disease, (3) prevalence of the virus, and (4) field reports suggest there may be a long-term or persistent infection with the potential for acute recrudescence of clinical disease post transport/stress.

The economic loss from the introduction of a foreign animal disease is multidimensional ranging from the acute direct losses to livestock producers to a chronic loss of production that can diminish an entire industry for extended periods of time, and perhaps permanently. In addition, the increased costs of food and reduced availability of once common products can lead to social instability. In 2018, African Swine Fever virus (ASFV) emerged for the first time in China, the largest producer of pork in the world accounting for over 50% of all production. African Swine Fever was first described in Africa in 1921 causing a fulminating disease in domestic swine (13). Wild swine found outside the continent of Africa are also quite susceptible, but in the African continent wild swine have co-evolved with ASFV developing some tolerance to the virus. Since its discovery, there have been sporadic regional epidemics in countries outside of Africa and only through heroic efforts could the virus be eradicated. In 2007, an ASFV epidemic began in Georgia in the Caucasus region that slowly, but steadily spread into Russia and then Europe (14). Although, the Chinese transmission event is unknown, this same lineage of virus jumped to China in mid-2018 and within 6 months had spread to all swine producing regions (15). The ASFV state-of-the-art control strategy is to depopulate the affected herd, and all pigs in potential contact with it. This strategy can be devastating in current production systems as occurred in Romania in August of 2018 when over 140,000 pigs were euthanized in an attempt to stop the spread of ASFV in that region (16). Currently, there is no vaccine for use in the control and prevention of ASFV which makes research in this area a top priority as the virus is now spreading in countries contiguous with China. An ASFV-positive status in just one pig (domestic or wild), immediately restricts the movement of domestic pigs and pork products within and from that country. Depending on regulatory infrastructure, some countries that have only had ASFV-infection in wild swine may have some export restrictions reduced, but the economic impact on disrupted markets is still substantial. The ASFV-pig interaction is very complicated and despite extensive research, the correlates of protection are poorly defined and current technology is inadequate to produce a safe and efficacious vaccine.

The constant need for more food in the world requires each agriculture commodity to be more efficiently produced which includes a perpetual battle against disease in crops and livestock. In the case of swine, the continual evolution of viruses requires sustained research into the basic immunology against these pathogens to provide new mechanistic insights into how pigs, and perhaps other species, respond to pathogens and enable the production of safer and more efficacious vaccines and anti-viral approaches. This knowledge may enable the application of novel technologies that could modulate the pig's immune response in the fight against disease, and ensure the availability of safe and wholesome pork around the globe. In response to the title, it is VERY important to study viral immunity in swine as well as livestock and poultry because more efficient production of meat protein is essential to the quest for more food.

## Author Contributions

KL and AB contributed to and wrote the manuscript.

### Conflict of Interest

The authors declare that the research was conducted in the absence of any commercial or financial relationships that could be construed as a potential conflict of interest.
